# Cascade radical reaction of substrates with a carbon–carbon triple bond as a radical acceptor

**DOI:** 10.3762/bjoc.9.128

**Published:** 2013-06-13

**Authors:** Hideto Miyabe, Ryuta Asada, Yoshiji Takemoto

**Affiliations:** 1 School of Pharmacy, Hyogo University of Health Sciences, Minatojima, Chuo-ku, Kobe 650-8530, Japan; 2 Graduate School of Pharmaceutical Sciences, Kyoto University, Yoshida, Sakyo-ku, Kyoto 606-8501, Japan

**Keywords:** cascade, cyclization, enantioselective, free radical, Lewis acid, radical

## Abstract

The limitation of hydroxamate ester as a chiral Lewis acid coordination moiety was first shown in an intermolecular reaction involving a radical addition and sequential allylation processes. Next, the effect of hydroxamate ester was studied in the cascade addition–cyclization–trapping reaction of substrates with a carbon–carbon triple bond as a radical acceptor. When substrates with a methacryloyl moiety and a carbon–carbon triple bond as two polarity-different radical acceptors were employed, the cascade reaction proceeded effectively. A high level of enantioselectivity was also obtained by a proper combination of chiral Lewis acid and these substrates.

## Introduction

**Figure 1:**
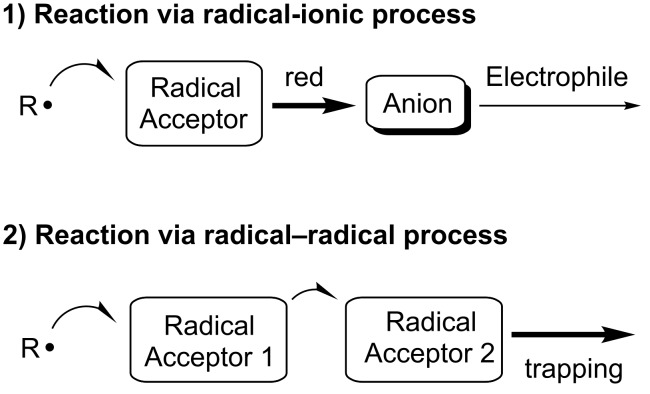
Cascade bond formation based on radical reactions.

Strategies involving a cascade process offer the advantage of multiple carbon–carbon and/or carbon–heteroatom bond formations in a single operation. Radical chemistry has been developed as one of the most powerful tools for carbon–carbon bond formation in organic synthesis [1–20]. Particularly, the advantages for utilizing the radical methodologies are the high functional group tolerance and the mild reaction conditions, because radical intermediates are not charged species. Therefore, a number of extensive investigations into sequential radical reactions have been reported over the past fifteen years and significant progress has been made in recent years [21–36]. We have also directed our efforts toward the development of new and efficient cascade approaches for the construction of carbon–carbon/heteroatom bonds based on radical chemistry. These approaches can be classified into two categories according to their reaction mechanism (Figure 1) [37–43].

**Figure 2:**
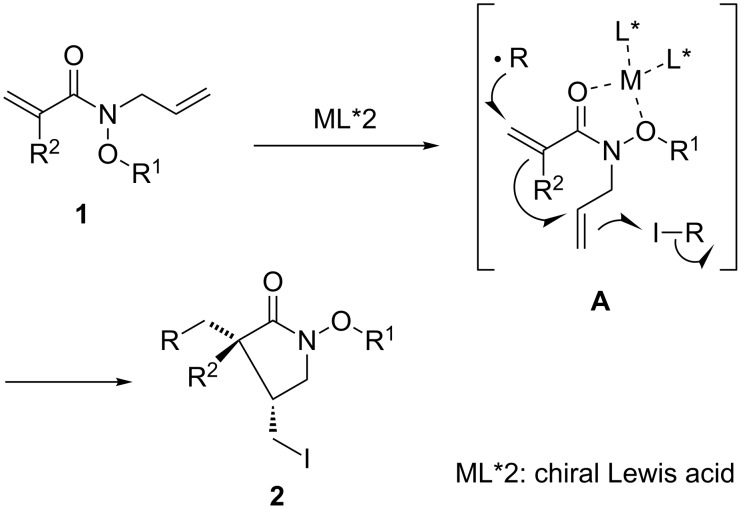
Our method for controlling the geometry of substrates and the stereochemistry of cyclization.

Enantioselective radical reactions have been intensively studied over the past fifteen years. Compared with stereocontrol studies on intermolecular radical reactions, the enantioselective stereocontrol in radical cyclizations still remains a major challenge [44–68]. We have also investigated a new type of chiral Lewis acid mediated cyclization approach for cascade bond-forming reactions via sequential radical–radical processes (Figure 2) [39–43]. In these studies, the control of the enantioselectivities was achieved by the introduction of a hydroxamate ester as a two-point-binding coordination tether into the middle of substrates **A**, together with the control of the rotamer population of substrates [39,42]. In this paper, we describe in detail the cascade addition–cyclization–trapping reaction of substrates with a carbon–carbon triple bond as a radical acceptor as well as the effect of hydroxamate ester as a Lewis acid coordination moiety. Some results have been reported in our preliminary communication [39].

## Results and Discussion

**Scheme 1:**
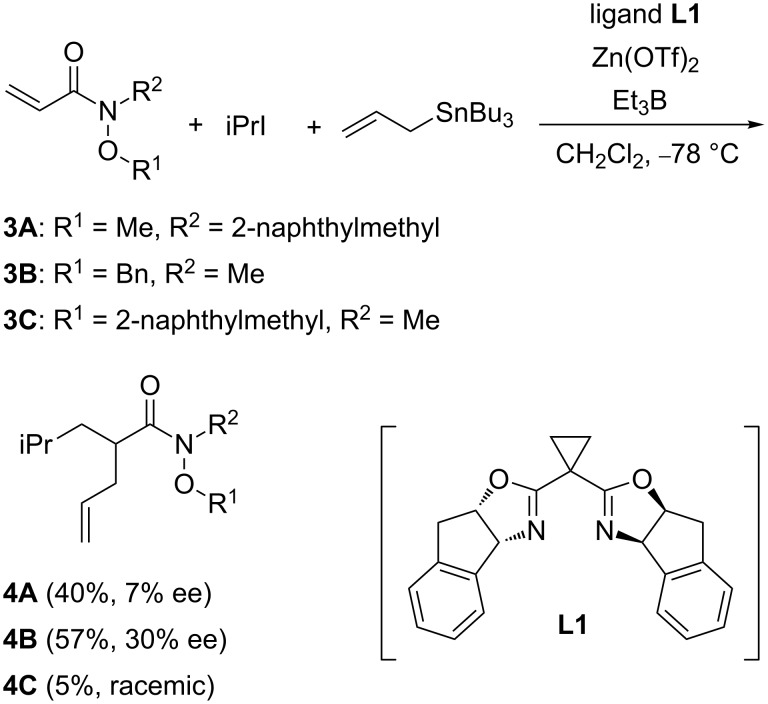
Effect of hydroxamate ester on intermolecular C–C bond-forming reactions.

Renaud’s group showed in 2002 that hydroxamic acid derivatives are useful achiral templates in enantioselective Diels–Alder reactions [69–70]. To study the effect of hydroxamate ester as an achiral template in the intermolecular radical reaction, our experiments began with the investigation of cascade radical addition–allylation of hydroxamate esters **3A**–**C** having an acryloyl moiety (Scheme 1). The reactions were evaluated in CH_2_Cl_2_ at −78 °C by employing isopropyl iodide, allyltin reagent, and Et_3_B as a radical initiator. The enantiomeric purities of products were checked by chiral HPLC analysis. The effect of the substituents R^1^ and R^2^ of hydroxamate esters **3A**–**C** on yield and selectivity was evaluated in the presence of a chiral Lewis acid prepared from box ligand **L1** and Zn(OTf)_2_. The results are shown in Scheme 1. Although good enantioselectivities were not observed, the size of the substituents had an impact on enantioselectivity with the larger group leading to lower ee. These observations indicate that the formation of the rigid ternary complex of hydroxamate ester, Zn(OTf)_2_ and the ligand **L1** is required for enantioselective transformation. A similar trend was observed in our studies on the addition–cyclization–trapping reaction of hydroxamate esters [39,42]. The chiral Lewis acid promoted the reaction of substrate **3A** having a bulky 2-naphthylmethyl group as substituent R^2^ to form the product **4A** in 40% yield with 7% ee. Moderate enantioselectivity was observed by employing the substrate **3B** having a benzyl group as R^1^ and a methyl group as R^2^. Particularly, the steric factor of the fluxional substituent R^1^ affected not only enantioselectivity but also the chemical efficiency. The use of **3C** having a 2-naphthylmethyl group as R^1^ led to a decrease in the chemical yield, probably because of the steric repulsion by a bulky substituent R^1^ leading to the dissociation of the chiral Lewis acid. In these studies, the absolute configuration at newly generated stereocenters has been not determined.

**Figure 3:**
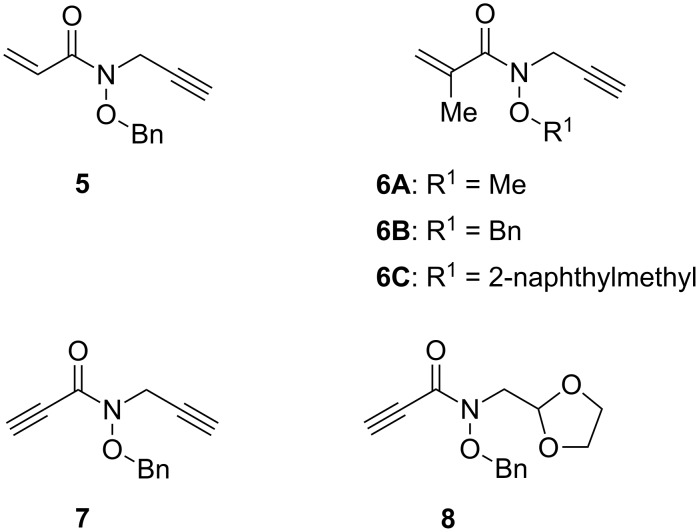
Substrates for testing the cascade transformation.

We recently reported in detail the cascade addition–cyclization–trapping reaction of substrates with carbon–carbon double bonds as two kinds of polarity-different radical acceptors [42]. On the basis of these results, the possibility of the carbon–carbon triple bond as a radical acceptor and the hydroxamate ester functionality as a two-point-binding coordination tether was next studied in detail. To understand the scope and limitation of the cascade transformation of hydroxamate esters with carbon–carbon triple bonds, the substrates of choice were **5**, **6A**–**C**, **7** and **8** having hydroxamate ester functionality (Figure 3).

**Scheme 2:**
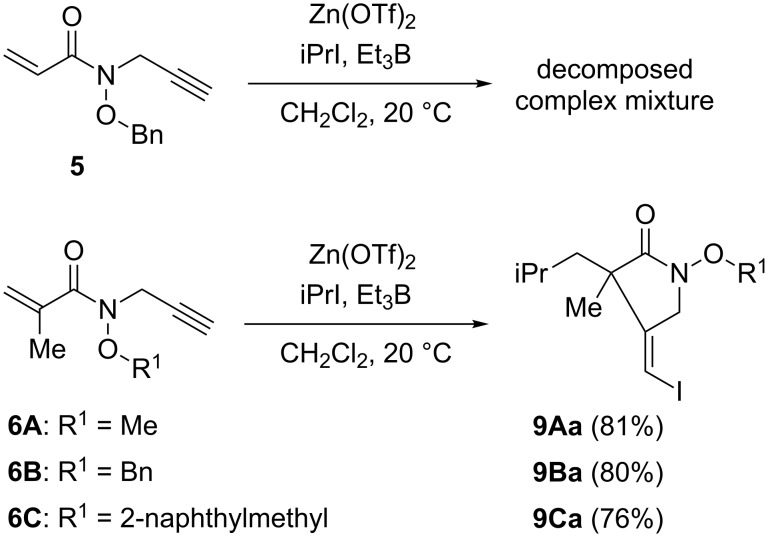
Cascade radical addition–cyclization–trapping reaction of **5** and **6A**–**C**.

At first, we studied the cascade reaction of **5** with an acryloyl moiety and **6A**–**C** with a methacryloyl moiety as an electron-deficient acceptor in the absence of a chiral ligand (Scheme 2). To control the rotamer population of substrates, Zn(OTf)_2_ was used as a Lewis acid to coordinate the hydroxamate ester functionality. The reactions were evaluated in CH_2_Cl_2_ at 20 °C under the tin-free iodine atom transfer conditions by using isopropyl iodide and Et_3_B. The reaction of hydroxamate ester **5** did not give the desired product probably due to polymerization of **5** through the labile acrylamide moiety. In contrast, the reaction of **6A**–**C** proceeded effectively to give the cyclic products **9Aa**–**9Ca** in good yields. Among them, hydroxamate esters **6A** and **6B**, which have a small methyl or benzyl group as R^1^, have shown a high reactivity, although a 76% yield of product **9Ca** was obtained even when hydroxamate ester **6C** having a 2-naphthylmethyl group was used. Furthermore, the regiochemical course of the initial radical addition to **6A**–**C** was well controlled. The nucleophilic isopropyl radical reacted selectively with the electron-deficient methacryloyl moiety to give the single isomers **9Aa**–**9Ca**.

**Figure 4:**
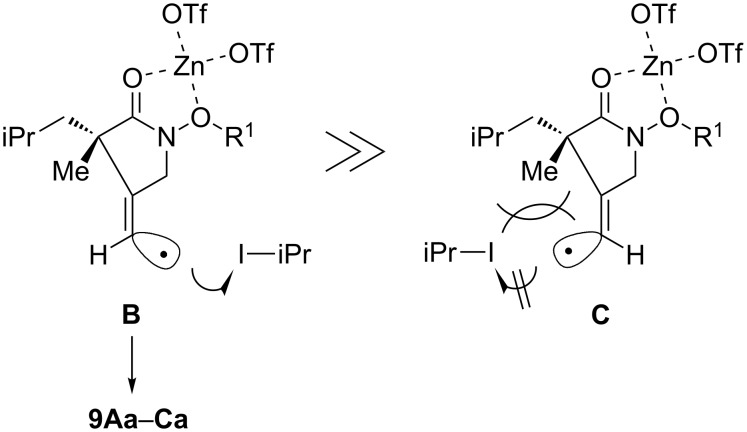
*E*/*Z*-selectivity of **9Aa**–**Ca**.

It is also important to note that *Z*-isomers **9Aa**–**9Ca** were selectively obtained without the formation of corresponding *E*-isomers. The *E*,*Z*-selectivities are determined by capturing the intermediate vinyl radicals with an atom-transfer reagent such as isopropyl iodide (Figure 4). These selectivities are controlled by the steric factor around vinyl radicals. The vinyl radicals are σ-radicals in a very fast equilibrium between *E*-isomer **B** and *Z*-isomer **C**. The steric hindrance between the substituents on the α-carbon atom of radical **C** and isopropyl iodide is assumed to lead to selective iodine atom-transfer in radical **B** giving **9Aa**–**9Ca** as single *Z*-isomers.

**Scheme 3:**
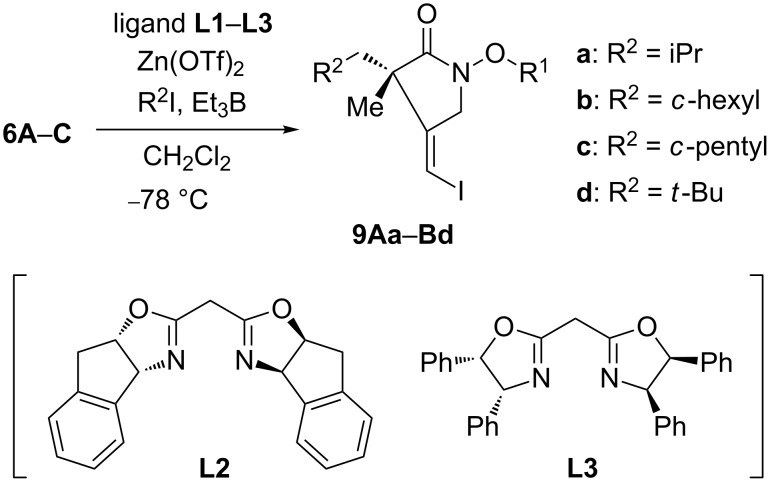
Enantioselective cascade reaction of **6A**–**C**.

On the basis of the above results, we next studied the reaction of **6A**–**C** at −78 °C in the presence of Zn(OTf)_2_ and chiral box ligands **L1**–**L3** (Scheme 3 and Table 1). A stoichiometric amount of chiral Lewis acid prepared from Zn(OTf)_2_ and ligand **L1** accelerated the reaction of hydroxamate ester **6A** having a methyl group as substituent R^1^ (Table 1, entry 1), although the reaction of **6A** did not proceed effectively at −78 °C in the absence of box ligand **L1**. The desired product **9Aa** was isolated as a single isomer in 51% yield with 60% ee after being stirred for 10 h. The use of hydroxamate ester **6B** having a benzyl group led to not only an enhancement in chemical yield but also to an improvement in enantioselectivity to give the product **9Ba** in 87% yield with 80% ee (Table 1, entry 2). Next, the catalytic nature of the reactions was examined (Table 1, entries 3–5). The reactions proceeded equally well with 50 and 30 mol % of chiral Lewis acid as with a stoichiometric amount (Table 1, entry 3 and 4). Further reduction of the chiral Lewis acid load to 10 mol % resulted in a decrease of both the chemical yield and enantioselectivity (Table 1, entry 5). In the case of 10 mol % of the chiral Lewis acid, the ternary complex of the ligand, the Lewis acid and the substrate were not effectively formed, and the background reaction giving the racemic product proceeded. Additionally, the high *Z*-selectivity of product **9Ba** indicates that the stereoselective iodine-atom transfer from isopropyl iodide to an intermediate radical proceeded effectively under these catalytic reaction conditions. The reaction using box ligand **L2** instead of **L1** attenuated the enantioselectivity (Table 1, entry 6). A somewhat lower enantioselectivity was obtained by using ligand **L3**, surprisingly resulting in antipode product **9Ba** (Table 1, entry 7). The representative effect of the solvent is shown in Table 1, entries 8–10. No reaction occurred in toluene, owing to the low solubility of the chiral Lewis acid in toluene (Table 1, entry 8). When the reaction was carried out in toluene/CH_2_Cl_2_ (4:1, v/v), the cyclic product **9Ba** was obtained in 67% yield with 77% ee (Table 1, entry 9). The reaction in the protic solvent MeOH gave the nearly racemic product, although the high *Z*-selectivity was maintained (Table 1, entry 10). These results suggest that the rigid chelation of the chiral Lewis acid to the hydroxamate ester functionality occured in CH_2_Cl_2_. In the presence of chiral Lewis acid, hydroxamate ester **6C** had also shown good reactivity, although the enantioselectivity diminished to 75% ee (Table 1, entry 11). We next studied the reaction of substrate **6B** with other radical precursors (Table 1, entries 12–14). Reactions with cyclohexyl and cyclopentyl radicals were also facile. Under analogous reaction conditions, an outstanding level of enantioselectivity was observed on employing the bulky *tert*-butyl iodide as a radical precursor (Table 1, entry 14). A good yield of the product **9Bd** was obtained with 92% ee and high *Z*-selectivity.

**Table 1:** Reaction of **6A**–**C** in the presence of chiral ligand.

entry	substrate	R^2^	ligand	Lewis acid (equiv)	product (% yield)	*Z*/*E*	ee (%)

1	**6A**	iPr	**L1**	1.0	**9Aa** (51)	>98:2	60
2 [39]	**6B**	iPr	**L1**	1.0	**9Ba** (87)	>98:2	80
3 [39]	**6B**	iPr	**L1**	0.5	**9Ba** (85)	>98:2	81
4 [39]	**6B**	iPr	**L1**	0.3	**9Ba** (82)	>98:2	81
5 [39]	**6B**	iPr	**L1**	0.1	**9Ba** (49)^a^	>98:2	47
6	**6B**	iPr	**L2**	1.0	**9Ba** (76)	>98:2	71
7	**6B**	iPr	**L3**	1.0	**9Ba** (81)	>98:2	–69
8^b^	**6B**	iPr	**L1**	1.0	no reaction		
9^c^	**6B**	iPr	**L1**	1.0	**9Ba** (67)	>98:2	77
10^d^	**6B**	iPr	**L1**	1.0	**9Ba** (63)	>98:2	rac
11	**6C**	iPr	**L1**	1.0	**9Ca** (83)	>98:2	75
12 [39]	**6B**	*c*-Hex	**L1**	1.0	**9Bb** (82)	>98:2	81
13	**6B**	*c*-Pent	**L1**	1.0	**9Bc** (83)	>98:2	79
14 [39]	**6B**	*t*-Bu	**L1**	1.0	**9Bd** (85)	>98:2	92

^a^starting substrate **6B** was recovered in 29% yield; ^b^in toluene; ^c^in toluene/CH_2_Cl_2_ (4:1, v/v); ^d^in MeOH.

**Figure 5:**
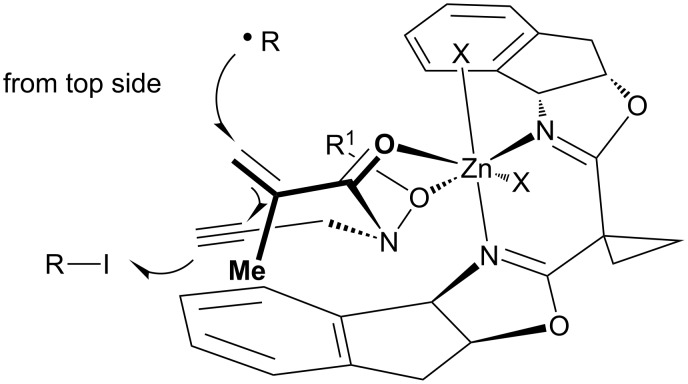
Model for the enantioselective reaction.

The absolute configuration at the newly generated stereocenters of **9Aa**–**Bd** was assumed by similarity between the present reaction and the previously reported reaction of substrates having the carbon–carbon double bond [39,42]. In these reactions, a ternary complex of ligand, Lewis acid and substrate would control the three-dimensional arrangement of two radical acceptors. A tetrahedral or *cis*-octahedral geometry around the zinc center was proposed [71–72]. In Figure 5, a tentative model of an octahedral complex is shown, in which two oxygen atoms of the hydroxamate ester functionality occupy two equatorial positions.

**Scheme 4:**
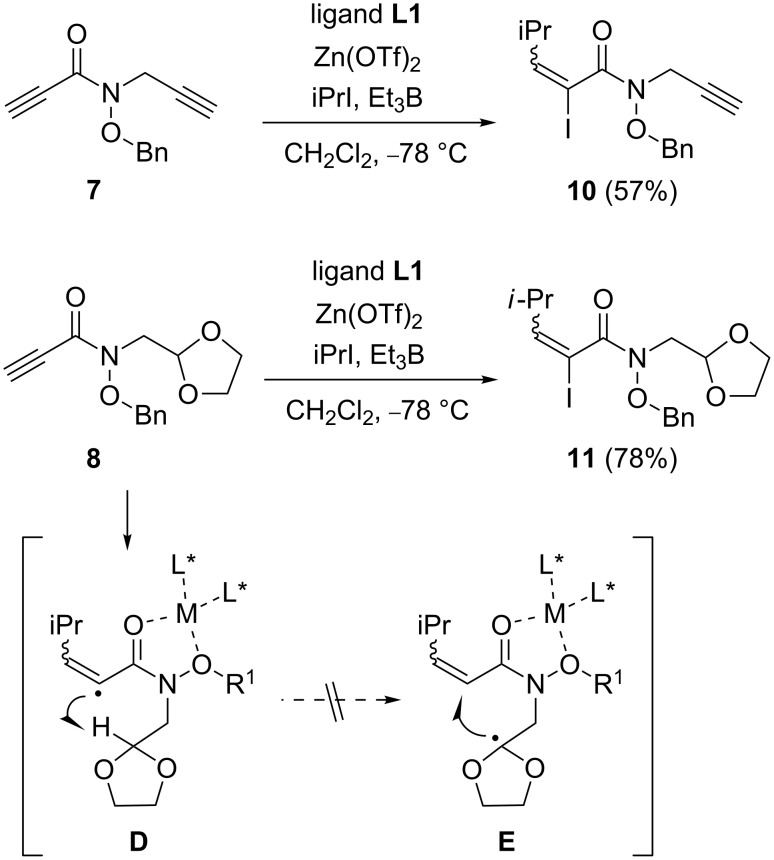
Reaction of propiolic acid derivatives **7** and **8**.

To study the effect of an electron-deficient acceptor on the cascade process, the reactions of propiolic acid derivatives **7** and **8** were tested (Scheme 4). At first, the reaction of **7** was evaluated under asymmetric reaction conditions. However, the cascade addition–cyclization–trapping reaction did not proceed, and the simple adduct **10** was formed in 57% yield by the addition–trapping process. Next, the reaction of propiolic acid derivative **8** was tested, because we expected the [1,5]-hydrogen shift from 1,3-dioxolane ring into the reactive vinyl radical as shown as **D**. However, the simple adduct **11** was only obtained in 78% yield. The results from these studies show that a carbon–carbon double bond, e.g., a methacryloyl group, of the electron-deficient acceptor is essential for the successful cascade transformation.

**Scheme 5:**
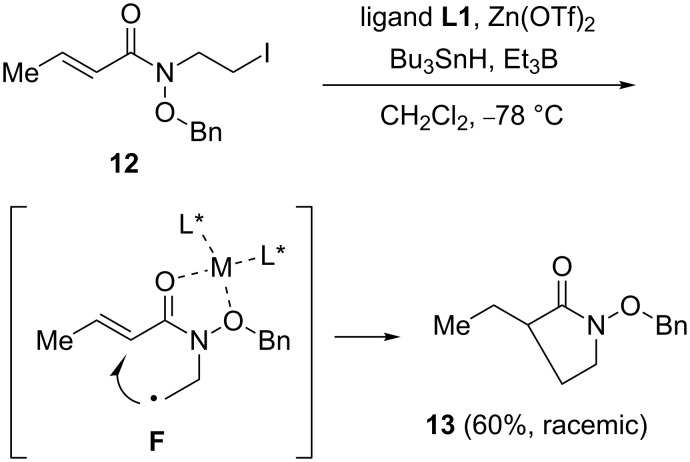
Opposite regiochemical cyclization using substrate **12**.

To gain further insight into the stereocontrol in the cyclization step, we next studied the opposite regiochemical cyclization by using the substrate **12** via the intermediate radical **F** (Scheme 5). The reaction was carried out in the presence of Bu_3_SnH under asymmetric reaction conditions. Although the reaction proceeded even at −78 °C, the nearly racemic product **13** was isolated in 60% yield. This observation indicates that the regiochemical course of the cyclization step is an important factor to achieve the highly asymmetric induction.

**Scheme 6:**
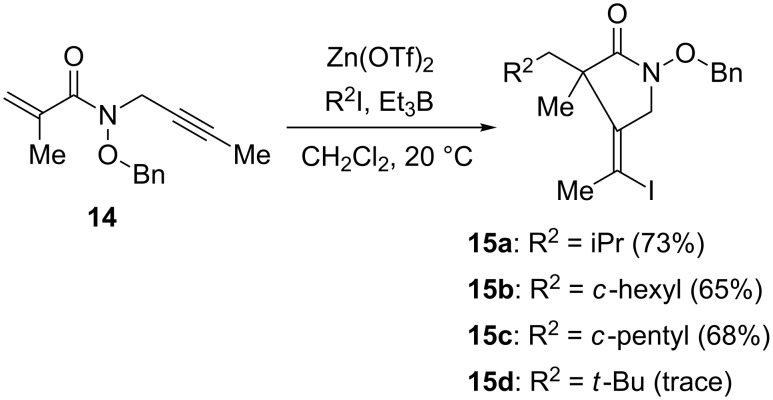
Cascade reaction of **14**.

We next investigated the reactivity of internal alkynes as electron-rich acceptors (Scheme 6). The internal alkyne **14** has shown a good reactivity comparable to that of the terminal alkynes **6A**–**C**. In the absence of a chiral ligand, the zinc Lewis acid accelerated the reaction of alkyne **14** with an isopropyl radical at 20 °C to give the desired cyclic product **15a** in 73% yield. Under analogous reaction conditions, both cyclohexyl iodide and cyclopentyl iodide worked well to give **15b** and **15c** in 65% and 68% yields, respectively. However, the reaction with a bulky *tert*-butyl radical did not proceed effectively, probably due to side reactions such as polymerization.

**Scheme 7:**
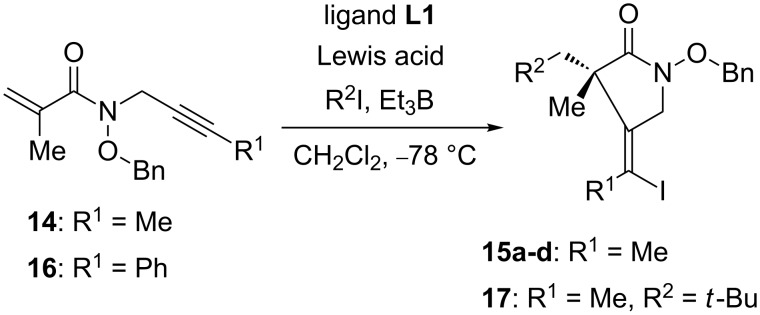
Enantioselective cascade reaction of **14** and **16**.

We finally investigated the enantioselective reaction of internal alkynes **14** and **16** (Scheme 7). The reaction of **14** proceeded with good enantioselectivities (Table 2). When a stoichiometric amount of chiral Lewis acid was employed, the reaction with an isopropyl radical gave the desired product **15a** in 86% yield with 83% ee (Table 2, entry 1). The reaction proceeded equally well with 30 mol % of chiral Lewis acid as with a stoichiometric amount (Table 2, entry 2). The secondary radicals, generated from cyclohexyl iodide or cyclopentyl iodide, reacted well to afford **15b** and **15c** with 85% ee and 83% ee, respectively (Table 2, entry 3 and 4). In marked contrast to the reaction in the absence of a chiral ligand (Scheme 6), the use of bulky *tert*-butyl iodide led to not only an enhancement in chemical yield but also to an improvement in enantioselectivity (Table 2, entry 5). These observations indicate that the combination of chiral Lewis acid and hydroxamate ester functionality led the rigid complex promoting the cyclization step and suppressing the background reaction or the undesired side reactions. High chemical yield and enantioselectivity were observed with 50 mol % of chiral Lewis acid (Table 2, entry 6), although further reduction of the catalyst load to 30 mol % resulted in a decrease of yield and enantioselectivity (Table 2, entry 7). Both chemical yield and enantioselectivity decreased by changing Lewis acid from Zn(OTf)_2_ to MgI_2_ (Table 2, entry 8). When the more nucleophilic and stable *tert*-butyl radical was employed, the reaction of substrate **16** having a phenyl group at the terminal position proceeded smoothly to give the desired product **17** in 89% yield with 67% ee (Table 2, entry 9). It is also important to note that the high *Z*/*E-*selectivity of products was observed even when internal alkynes **14** and **16** were employed. These results indicate that the iodine atom-transfer from R^2^I to the substituted vinyl radicals proceeded stereoselectively. Particularly, the substrate **16** having a phenyl group gave the intermediate linear π-radical. Thus, the capture of linear vinyl radical with atom-transfer reagent would be influenced by the steric hindrance around the quaternary carbon atom [43].

**Table 2:** Reaction of **14** and **16** in the presence of a chiral ligand.

entry	substrate	R^2^	Lewis acid (equiv)	yield (%)	ratio	ee (%)

1 [39]	**14**	iPr	Zn(OTf)_2_ (1.0)	86	>98:2	83
2 [39]	**14**	iPr	Zn(OTf)_2_ (0.3)	74	>98:2	81
3 [39]	**14**	*c*-Hex	Zn(OTf)_2_ (1.0)	87	>98:2	85
4	**14**	*c*-Pent	Zn(OTf)_2_ (1.0)	77	>98:2	83
5 [39]	**14**	*t*-Bu	Zn(OTf)_2_ (1.0)	94	>98:2	90
6	**14**	*t*-Bu	Zn(OTf)_2_ (0.5)	94	>98:2	91
7	**14**	*t*-Bu	Zn(OTf)_2_ (0.3)	75	>98:2	61
8	**14**	*t*-Bu	MgI_2_ (1.0)	20	>98:2	54
9	**16**	*t*-Bu	Zn(OTf)_2_ (1.0)	89	>98:2	67

## Conclusion

We have shown the cascade radical addition–cyclization–trapping reaction of substrates with a carbon–carbon triple bond as a radical acceptor as well as the scope and limitation of hydroxamate ester as a coordination site with a chiral Lewis acid. Synthetic strategies involving enantioselective radical cyclizations would be desirable tools for preparing functionalized cyclic compounds with multiple stereocenters. These studies offer opportunities for further exploration of fascinating possibilities in the realm of cascade radical reactions.

## Supporting Information

File 1 General experimental procedures, characterization data of obtained compounds, and preparation of substrates.
