# Supplementary material for: Cascade radical reaction of substrates with a carbon–carbon triple bond as a radical acceptor
Source: Beilstein J Org Chem. 2013 Jun 13;9:1148–55. doi: 10.3762/bjoc.9.128 (PMC3701377; doi:10.3762/bjoc.9.128)
Supplement: File 1 — General experimental procedures, characterization data of obtained compounds, and preparation of substrates. [file Beilstein_J_Org_Chem-09-1148-s001.pdf]

# Supporting Information

## for

## Cascade radical reaction of substrates with a carbon–carbon triple bond as a radical acceptor

Hideto Miyabe*[1,2], Ryuta Asada[2] and Yoshiji Takemoto*[2]

[1]School of Pharmacy, Hyogo University of Health Sciences, Minatojima, Chuo-ku, Kobe 650-8530, Japan and [2]Graduate School of Pharmaceutical Sciences, Kyoto University, Yoshida, Sakyo-ku, Kyoto 606-8501, Japan

Email: Hideto Miyabe* - miyabe@huhs.ac.jp

*Corresponding author

## General experimental procedures, characterization data of obtained compounds, and preparation of substrates

Contents

1. Experimental procedure
2. Characterization data of obtained compounds
3. Preparation of substrates
4. References

**1. Experimental procedures**

The chiral ligand **L1** was prepared according to the reported method [1]. The commercially available chiral ligands **L2** and **L3** were used without further purification.

**General procedure for the radical reaction.** In a similar manner as reported in [2], a solution of substrates **5**, **6A–C** or **14** (0.5 mmol) and $Zn(OTf)_2$ (182 mg, 0.5 mmol) in $CH_2Cl_2$ (3 mL) was stirred for 30 min under a nitrogen atmosphere at 20 °C. To this reaction mixture were added RI (15 mmol) and $Et_3B$ (1.0 M in hexane, 1.25 mL, 1.25 mmol) at 20 °C. After being stirred at the same temperature for 5–10 h, the reaction mixture was diluted with saturated $NaHCO_3$ and then extracted with AcOEt. The organic phase was dried over $MgSO_4$ and concentrated under reduced pressure. Purification of the residue by column chromatography (hexane/AcOEt 4:1) afforded products **9Aa–Ca** or **15a–d**.

**General procedure for the enantioselective radical reaction.** In a similar manner as reported in [3], a solution of substrates **3A–C**, **6A–C**, **7**, **8**, **14** or **16** (0.5 mmol), Lewis acid (0.5–0.05 mmol) and ligand (0.5–0.05 mmol) in $CH_2Cl_2$ (3 mL) was stirred for 30 min under a nitrogen atmosphere at 20 °C. To this reaction mixture were added RI (15 mmol) and $Et_3B$ (1.0 M in hexane, 1.25 mL, 1.25 mmol) at −78 °C. After being stirred at the same temperature for 3–10 h, the reaction mixture was diluted with saturated $NaHCO_3$ and then extracted with AcOEt. The organic phase was dried over $MgSO_4$ and concentrated under reduced pressure. Purification of the residue by column chromatography (hexane/AcOEt 4:1) afforded products **4A–C**, **9Aa**–**Bd**, **10**, **11**, **15a**–**d** or **17**.

**Procedure for radical cyclization of 12.** A solution of substrate **12** (173 mg, 0.5 mmol), $Zn(OTf)_2$ (182 mg, 0.5 mmol) and ligand **L1** (178 mg, 0.5 mmol) in $CH_2Cl_2$ (3 mL) was stirred for 30 min under a nitrogen atmosphere at 20 °C. To this reaction mixture was added $Et_3B$ (1.0 M in hexane, 1.25 mL, 1.25 mmol) at −78 °C. After being stirred at the same temperature for 7 h, the reaction mixture was diluted with saturated $NaHCO_3$ and then extracted with AcOEt. The organic phase was dried over $MgSO_4$ and concentrated under reduced pressure. Purification of the residue by column chromatography (hexane/AcOEt 10:1) afforded product **13** (66 mg, 60%).

**2. Characterization data of compounds**

The characterization data of **9Ba**, **9Bb**, **9Bd**, **15a**, **15b** and **15d** were reported in our previously published article [3].

**Allylated product (4A).** Colorless oil; IR ($CHCl_3$) 1650 $cm^{-1}$; $^1H$ NMR ($CDCl_3$) δ 7.84-7.76 (4H, m), 7.49-7.42 (3H, m), 5.76 (1H, m), 5.06 (1H, dd, *J* = 17.1, 1.5 Hz), 5.00-4.90 (3H, m), 3.64 (3H, s), 3.03 (1H, br m), 2.39 (1H, m), 2.19 (1H, m), 1.68-1.52 (2H, m), 1.30 (1H, m), 0.89 (3H, d, *J* = 6.4 Hz), 0.86 (3H, d, *J* = 6.4 Hz); $^{13}C$ NMR ($CDCl_3$) δ 177.3, 136.2, 134.3, 133.3, 132.9, 128.3, 127.8, 127.7, 127.5, 126.5, 126.2, 125.9, 116.6, 62.4, 49.1, 41.2, 38.8, 36.9, 25.9, 22.9, 22.5; MS ($EI^+$): *m/z* 325 ($M^+$, 0.2), 69 (100); HRMS ($EI^+$): calcd for $C_{21}H_{27}NO_2$ ($M^+$): 325.2041, found: 325.2044; HPLC (Chiralcel OJ-H, hexane/2-propanol 98:2, 1.0 mL/min, 254 nm) $t_r$ (minor) = 7.5 min, $t_r$ (major) = 8.3 min.

**Allylated product (4B).** Colorless oil; IR ($CHCl_3$) 1646 $cm^{-1}$; $^1H$ NMR ($CDCl_3$) δ 7.43-7.35 (5H, m), 5.72 (1H, m), 5.05 (1H, br d, *J* = 17.1 Hz), 5.00 (1H, br d, *J* = 10.0 Hz), 4.85 (1H, d, *J* = 10.3 Hz), 4.83 (1H, d, *J* = 10.3 Hz), 3.20 (3H, s), 3.06 (1H, br m), 2.30 (1H, m), 2.16 (1H, m), 1.63-1.49 (2H, m), 1.28 (1H, m), 0.87 (3H, d, *J* = 6.4 Hz), 0.85 (3H, d, *J* = 6.4 Hz); $^{13}C$ NMR ($CDCl_3$) δ 177.2, 136.3, 134.6, 129.2, 128.9, 128.7, 116.5, 76.5, 41.1, 38.9, 36.8, 33.6, 25.9, 22.8, 22.6; MS ($EI^+$): *m/z* 275 ($M^+$, 0.5), 61 (100); HRMS ($EI^+$): calcd for $C_{17}H_{25}NO_2$ ($M^+$): 275.1885, found: 275.1890; HPLC (Chiralcel OJ-H, hexane/2-propanol 99:1, 0.3 mL/min, 254 nm) $t_r$ (minor) = 33.6 min, $t_r$ (major) = 39.0 min. A sample of 30% ee by HPLC analysis gave $[\alpha]^{24}_D$ −2.3 (*c* 0.9, $CHCl_3$).

**Allylated product (4C).** Colorless oil; IR ($CHCl_3$) 1645 $cm^{-1}$; $^1H$ NMR ($CDCl_3$) δ 7.90-7.80 (4H, m), 7.54-7.46 (3H, m), 5.71 (1H, m), 5.08-4.93 (4H, m), 3.23 (3H, s), 3.10 (1H, br m), 2.33 (1H, m), 2.17 (1H, m), 1.65-1.49 (2H, m), 1.29 (1H, m), 0.86 (6H, m); $^{13}C$ NMR ($CDCl_3$) δ 178.0, 136.3, 133.4, 133.2, 132.0, 128.6, 128.5, 128.1, 127.8, 126.6, 126.5 (2C), 116.5, 76.7, 41.2, 39.0, 36.8, 33.8, 26.0, 22.8, 22.6; MS ($FAB^+$): *m/z* 326 (M + $H^+$, 91), 141 (100); HRMS ($FAB^+$): calcd for $C_{21}H_{28}NO_2$ (M + $H^+$): 326.2120, found: 326.2122; HPLC (Chiralcel OJ-H, hexane/2-propanol 95:5, 0.5 mL/min, 254 nm) $t_r$ (racemic) = 21.0 and 22.8 min.

**Cyclic product (9Aa).** Colorless oil; IR ($CHCl_3$) 1713 $cm^{-1}$; $^1H$ NMR ($CDCl_3$) δ 6.22 (1H, t, *J* = 2.8 Hz), 4.07 (1H, dd, *J* = 14.1, 2.8 Hz), 4.02 (1H, dd, *J* = 14.1, 2.8 Hz), 3.85 (3H, s), 1.80 (1H, dd, *J* = 13.8, 7.4 Hz), 1.56 (1H, m), 1.49 (1H, dd, *J* = 13.8, 5.2 Hz), 1.26 (3H, s), 0.84 (2H, d, *J* = 6.7 Hz), 0.82 (3H, d, *J* = 7.6 Hz); $^{13}C$ NMR ($CDCl_3$) δ 173.0, 147.8, 74.6, 62.0, 53.8, 49.8, 47.7, 26.9, 25.3, 24.3, 22.9; MS ($CI^+$): *m/z* 324 (M + $H^+$, 12), 267 (100); HRMS ($CI^+$): calcd for $C_{11}H_{19}INO_2$ (M + $H^+$): 324.0461, found: 324.0463; HPLC (Chiralcel AD-H, hexane/2-propanol 95:5, 1.0 mL/min, 254 nm) $t_r$ (major) = 6.2 min, $t_r$ (minor) = 6.9 min. A sample of 60% ee by HPLC analysis gave $[\alpha]^{28}_D$ −3.6 (*c* 1.1, $CHCl_3$).

**Cyclic product (9Ca).** Colorless oil; IR ($CHCl_3$) 1714 $cm^{-1}$; $^1H$ NMR ($CDCl_3$) δ 7.91-7.80 (4H, m), 7.62 (1H, br d, *J* = 8.2 Hz), 7.52-7.47 (2H, m), 6.11 (1H, t, *J* = 2.7 Hz), 5.23 (1H, d, *J* = 11.3 Hz), 5.20 (1H, d, *J* = 11.3 Hz), 3.84 (2H, d, *J* = 2.7 Hz), 1.75 (1H, dd, *J* = 13.1, 5.8 Hz), 1.48-1.35 (2H, m), 1.21 (3H, s), 0.77 (3H, d, *J* = 6.4 Hz), 0.72 (3H, d, *J* = 6.4 Hz); $^{13}C$ NMR ($CDCl_3$) δ 173.4, 147.9, 133.5, 133.0, 132.3, 128.9, 128.5, 128.1, 127.8, 126.6, 126.5, 126.4, 76.9,

74.3, 55.4, 49.8, 47.5, 26.9, 25.3, 24.0, 23.2; MS ($FAB^+$): *m/z* 450 ($M + H^+$, 10), 141 (100); HRMS ($FAB^+$): calcd for $C_{21}H_{25}INO_2$ ($M + H^+$): 450.0931, found: 450.0927; HPLC (Chiralcel AD-H, hexane/2-propanol 95:5, 1.0 mL/min, 254 nm) $t_r$ (major) = 12.6 min, $t_r$ (minor) = 20.0 min. A sample of 75% ee by HPLC analysis gave $[\alpha]^{26}_D$ +7.8 (*c* 0.8, $CHCl_3$).

**Cyclic product (9Bc).** Colorless oil; IR ($CHCl_3$) 1712 $cm^{-1}$; $^1H$ NMR ($CDCl_3$) δ 7.49-7.33 (5H, m), 6.14 (1H, t, *J* = 2.5 Hz), 5.06 (1H, d, *J* = 10.9 Hz), 5.04 (1H, d, *J* = 10.9 Hz), 3.78 (2H, m), 1.91 (1H, m), 1.65-1.25 (8H, m), 1.23 (3H, s), 1.13-0.91 (2H, s); $^{13}C$ NMR ($CDCl_3$) δ 173.5, 148.2, 135.0, 129.5, 129.1, 128.6, 76.9, 74.4, 55.6, 50.2, 45.3, 37.1, 33.8, 33.2, 26.3, 24.9, 24.8; MS ($FAB^+$): *m/z* 426 ($M + H^+$, 54), 91 (100); HRMS ($FAB^+$): calcd for $C_{19}H_{25}INO_2$ ($M + H^+$): 426.0930, found: 426.0935; HPLC (Chiralcel AD-H, hexane/2-propanol 95:5, 1.0 mL/min, 254 nm) $t_r$ (major) = 11.2 min, $t_r$ (minor) = 16.9 min. A sample of 79% ee by HPLC analysis gave $[\alpha]^{26}_D$ −3.3 (*c* 0.8, $CHCl_3$).

**Adduct (10).** Major isomer: Colorless oil; IR ($CHCl_3$) 1656 $cm^{-1}$; $^1H$ NMR ($CDCl_3$) δ 7.44-7.32 (5H, m), 6.01 (1H, d, *J* = 8.9 Hz), 4.99 (2H, s), 4.35 (1H, d, *J* = 2.5 Hz), 2.63 (1H, m), 2.32 (1H, t, *J* = 2.5 Hz), 1.05 (6H, d, *J* = 6.7 Hz); $^{13}C$ NMR ($CDCl_3$) δ 167.4, 149.6, 134.3, 129.4, 129.0, 128.6, 88.6, 77.6, 72.7, 38.8, 35.8, 20.9. One peak of $^{13}C$ NMR was missing due to an overlap; MS ($EI^+$): *m/z* 383 ($M^+$, 2), 91 (100); HRMS ($EI^+$): calcd for $C_{16}H_{18}INO_2$ ($M^+$): 383.0382, found: 383.0377.

**Adduct (11).** Major isomer: Colorless oil; IR ($CHCl_3$) 1651 $cm^{-1}$; $^1H$ NMR ($CDCl_3$) δ 7.42-7.37 (5H, m), 5.99 (1H, d, *J* = 8.9 Hz), 5.21 (1H, t, *J* = 4.6 Hz), 4.89 (2H, s), 4.00 (2H, m), 3.90 (2H, m), 3.74 (2H, br d, *J* = 4.6 Hz), 2.62 (1H, m), 1.04 (6H, d, *J* = 6.7 Hz); $^{13}C$ NMR ($CDCl_3$) δ 167.8, 149.4, 134.4, 129.4, 128.9, 128.6, 100.8, 89.3, 76.6, 65.0, 51.4, 35.7, 20.9; MS ($FAB^+$): *m/z* 432 ($M + H^+$, 100); HRMS ($FAB^+$): calcd for $C_{17}H_{23}INO_4$ ($M + H^+$): 432.0672, found: 432.0671. Minor isomer: Colorless oil; IR ($CHCl_3$) 1652 $cm^{-1}$; $^1H$ NMR ($CDCl_3$) δ 7.50-7.33 (5H, m), 6.21 (1H, d, *J* = 10.6 Hz), 5.23 (1H, br s), 4.97 (2H, br s), 4.00 (2H, br m), 3.90 (2H, br m), 3.81 (2H, br m), 2.52 (1H, m), 0.98 (6H, d, *J* = 6.4 Hz); MS ($FAB^+$): *m/z* 432 ($M + H^+$, 83), 154 (100); HRMS ($FAB^+$): calcd for $C_{17}H_{23}INO_4$ ($M + H^+$): 432.0672, found: 432.0676.

**Cyclic product (13).** Colorless oil; IR ($CHCl_3$) 1671 $cm^{-1}$; $^1H$ NMR ($CDCl_3$) δ 7.43-7.24 (5H, m), 4.99 (1H, d, *J* = 11.0 Hz), 4.98 (1H, d, *J* = 11.0 Hz), 4.34 (1H, m), 4.21 (1H, q, *J* = 8.6 Hz), 2.69 (1H, m), 2.25 (1H, m), 1.80 (2H, m), 1.45 (1H, m), 0.96 (3H, t, *J* = 7.6 Hz); $^{13}C$ NMR ($CDCl_3$) δ 161.4, 137.9, 128.4, 128.2, 127.6, 76.1, 70.3, 40.5, 29.6, 24.8, 11.5; MS ($FAB^+$): *m/z* 220 ($M + H^+$, 100); HRMS ($FAB^+$): calcd for $C_{13}H_{18}NO_2$ ($M + H^+$): 220.1338, found: 220.1341; HPLC (Chiralcel OD-H, hexane/2-propanol 95:5, 0.5 mL/min, 254 nm) $t_r$ (racemic) = 26.1 and 52.1 min.

**Cyclic product (15c).** Colorless oil; IR ($CHCl_3$) 1710 $cm^{-1}$; $^1H$ NMR ($CDCl_3$) δ 7.49-7.34 (5H, m), 5.06 (1H, d, *J* = 10.7 Hz), 5.02 (1H, d, *J* = 10.7 Hz), 3.75 (2H, q, *J* = 2.1 Hz), 2.60 (3H, t, *J* = 2.1 Hz), 1.96 (1H, dd, *J* = 14.1, 7.0 Hz), 1.82 (1H, dd, *J* = 14.1, 6.1 Hz), 1.65-1.25 (7H, m), 1.33 (3H, s), 1.09-0.95 (2H, m); $^{13}C$ NMR ($CDCl_3$) δ 174.1, 138.5,

134.9, 129.5, 129.0, 128.6, 96.0, 76.7, 58.7, 49.6, 42.8, 37.6, 33.1, 33.0, 30.1, 24.9, 24.8, 24.5; MS ($FAB^+$): *m/z* 440 ($M + H^+$, 78), 91 (100); HRMS ($FAB^+$): calcd for $C_{20}H_{27}INO_2$ ($M + H^+$): 440.1086, found: 440.1081; HPLC (Chiralcel AD-H, hexane/2-propanol 95:5, 1.0 mL/min, 254 nm) $t_r$ (major) = 9.5 min, $t_r$ (minor) = 13.2 min. A sample of 83% ee by HPLC analysis gave $[\alpha]^{28}_D$ −3.1 (*c* 0.7, $CHCl_3$).

**Cyclic product (17).** Colorless oil; IR ($CHCl_3$) 1715 $cm^{-1}$; $^1$H NMR ($CDCl_3$) δ 7.51-7.48 (2H, m), 7.43-7.38 (3H, m), 7.36-7.23 (5H, m), 5.12 (1H, d, *J* = 10.7 Hz), 5.06 (1H, d, *J* = 10.7 Hz), 4.03 (1H, d, *J* = 14.4 Hz), 3.93 (1H, d, *J* = 14.4 Hz), 1.54 (1H, d, *J* = 14.7 Hz), 1.36 (1H, d, *J* = 14.7 Hz), 1.06 (3H, s), 0.89 (9H, s); $^{13}$C NMR ($CDCl_3$) δ 174.3, 142.8, 142.2, 135.0, 129.5, 129.1, 128.6, 128.5, 128.4, 128.0, 96.8, 76.8, 59.8, 50.4, 49.0, 31.5, 30.8, 28.1; MS ($FAB^+$): *m/z* 490 ($M + H^+$, 66), 91 (100); HRMS ($FAB^+$): calcd for $C_{24}H_{29}INO_2$ ($M + H^+$): 490.1243, found: 490.1246; HPLC (Chiralcel AD-H, hexane/2-propanol 95:5, 1.0 mL/min, 254 nm) $t_r$ (major) = 6.9 min, $t_r$ (minor) = 10.5 min. A sample of 67% ee by HPLC analysis gave $[\alpha]^{28}_D$ −1.9 (*c* 0.7, $CHCl_3$).

## 3. Preparation of substrates

Substrates **3A–B** were prepared as shown in Scheme 1. The preparation of starting materials **18A**, **18B** and **18C** and characterization data were reported in our previously published article [2]. **19B** is a known compound [5,6].

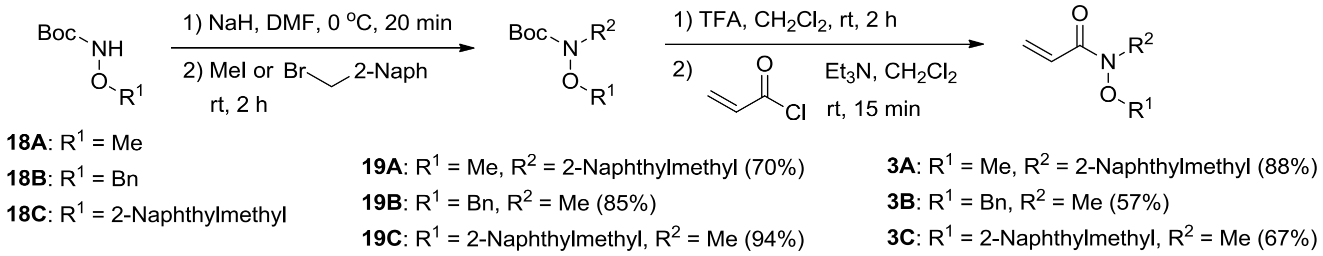


**Scheme 1.**

**(19A).** Colorless oil; IR ($CHCl_3$) 1700 $cm^{-1}$; $^1$H NMR ($CDCl_3$) δ 7.81-7.72 (4H, m), 7.48-7.37 (3H, m), 4.73 (2H, s), 3.56 (3H, s), 1.48 (9H, s); $^{13}$C NMR ($CDCl_3$) δ 156.2, 134.4, 133.1, 132.7, 128.0, 127.6, 127.5, 127.0, 126.2, 125.9, 125.7, 81.3, 62.3, 53.1, 28.0; MS ($EI^+$): *m/z* 287 ($M^+$, 2), 141 (100); HRMS ($EI^+$): calcd for $C_{17}H_{21}NO_3$ ($M^+$): 287.1521, found: 287.1516.

**(19B).** Colorless oil; IR ($CHCl_3$) 1695 $cm^{-1}$; $^1$H NMR ($CDCl_3$) δ 7.43-7.32 (5H, m), 4.83 (2H, s), 3.05 (3H, s), 1.50 (9H, s); $^{13}$C NMR ($CDCl_3$) δ 156.6, 135.4, 129.1, 128.1 (2C), 80.7, 76.1, 36.4, 27.9; MS ($FAB^+$): *m/z* 238 ($M + H^+$, 20), 91 (100); HRMS ($FAB^+$): calcd for $C_{13}H_{20}NO_3$ ($M + H^+$): 238.1443, found: 238.1437.

**(19C).** Colorless oil; IR ($CHCl_3$) 1695 $cm^{-1}$; $^1$H NMR ($CDCl_3$) δ 7.80-7.72 (4H, m), 7.46 (1H, br d, *J* = 8.6 Hz), 7.38 (2H, m), 4.90 (2H, s), 2.97 (3H, s), 1.41 (9H, s); $^{13}$C NMR ($CDCl_3$) δ 157.0, 133.3, 133.2 (2C), 128.5, 128.1, 128.0,

127.7, 126.9, 126.2, 126.1, 81.2, 76.5, 36.4, 28.2; MS ($FAB^+$): *m/z* 288 ($M + H^+$, 2), 141 (100); HRMS ($FAB^+$): calcd for $C_{17}H_{22}NO_3$ ($M + H^+$): 288.1599, found: 288.1609.

**(3A).** Colorless oil; IR ($CHCl_3$) 1653 $cm^{-1}$; $^1H$ NMR ($CDCl_3$) δ 7.85-7.76 (4H, m), 7.50-7.42 (3H, m), 6.77 (1H, dd, *J* = 17.1, 10.4 Hz), 6.53 (1H, m), 5.81 (1H, br d, *J* = 10.4 Hz), 5.02 (2H, s), 3.62 (3H, s); $^{13}C$ NMR ($CDCl_3$) δ 166.6, 133.9, 133.3, 132.9, 129.7, 128.4, 127.8, 127.7, 127.4, 126.3, 126.2, 126.1, 126.0, 62.9, 49.5; MS ($EI^+$): *m/z* 241 ($M^+$, 0.5), 141 (100); HRMS ($EI^+$): calcd for $C_{15}H_{15}NO_2$ ($M^+$): 241.1102, found: 241.1108.

**(3B).** Colorless oil; IR ($CHCl_3$) 1652 $cm^{-1}$; $^1H$ NMR ($CDCl_3$) δ 7.45-7.34 (5H, m), 6.72 (1H, dd, *J* = 17.1, 10.4 Hz), 6.40 (1H, dd, *J* = 17.1, 1.8 Hz), 5.71 (1H, dd, *J* = 10.4, 1.8 Hz), 4.85 (2H, s), 3.27 (3H, s); $^{13}C$ NMR ($CDCl_3$) δ 166.8, 134.1, 129.1, 128.8, 128.6, 128.5, 126.0, 76.7, 33.4; MS ($EI^+$): *m/z* 191 ($M^+$, 3), 77 (100); HRMS ($EI^+$): calcd for $C_{11}H_{13}NO_2$ ($M^+$): 191.0947, found: 191.0944.

**(3C).** Colorless oil; IR ($CHCl_3$) 1652 $cm^{-1}$; $^1H$ NMR ($CDCl_3$) δ 7.89-7.82 (4H, m), 7.59-7.45 (3H, m), 6.77 (1H, dd, *J* = 16.8, 10.7 Hz), 6.41 (1H, dd, *J* = 16.8, 1.8 Hz), 5.71 (1H, dd, *J* = 10.7, 1.8 Hz), 5.00 (2H, s), 3.28 (3H, s); $^{13}C$ NMR ($CDCl_3$) δ 167.1, 133.4, 133.1, 131.7, 129.0, 128.6 (2C), 128.1, 127.8, 126.7, 126.5, 126.4, 126.1, 77.1, 33.8; MS ($FAB^+$): *m/z* 242 ($M + H^+$, 61), 154 (100); HRMS ($FAB^+$): calcd for $C_{15}H_{16}NO_2$ ($M + H^+$): 242.1181, found: 242.1178.

Substrates **5**, **6A–C**, **7** and **8** were prepared as shown in Scheme 2. The characterization data of **6B** were reported in our previously published article [3]. The characterization data of **20B** were reported in our previously published article [4]. **20A** is a known compound [7].

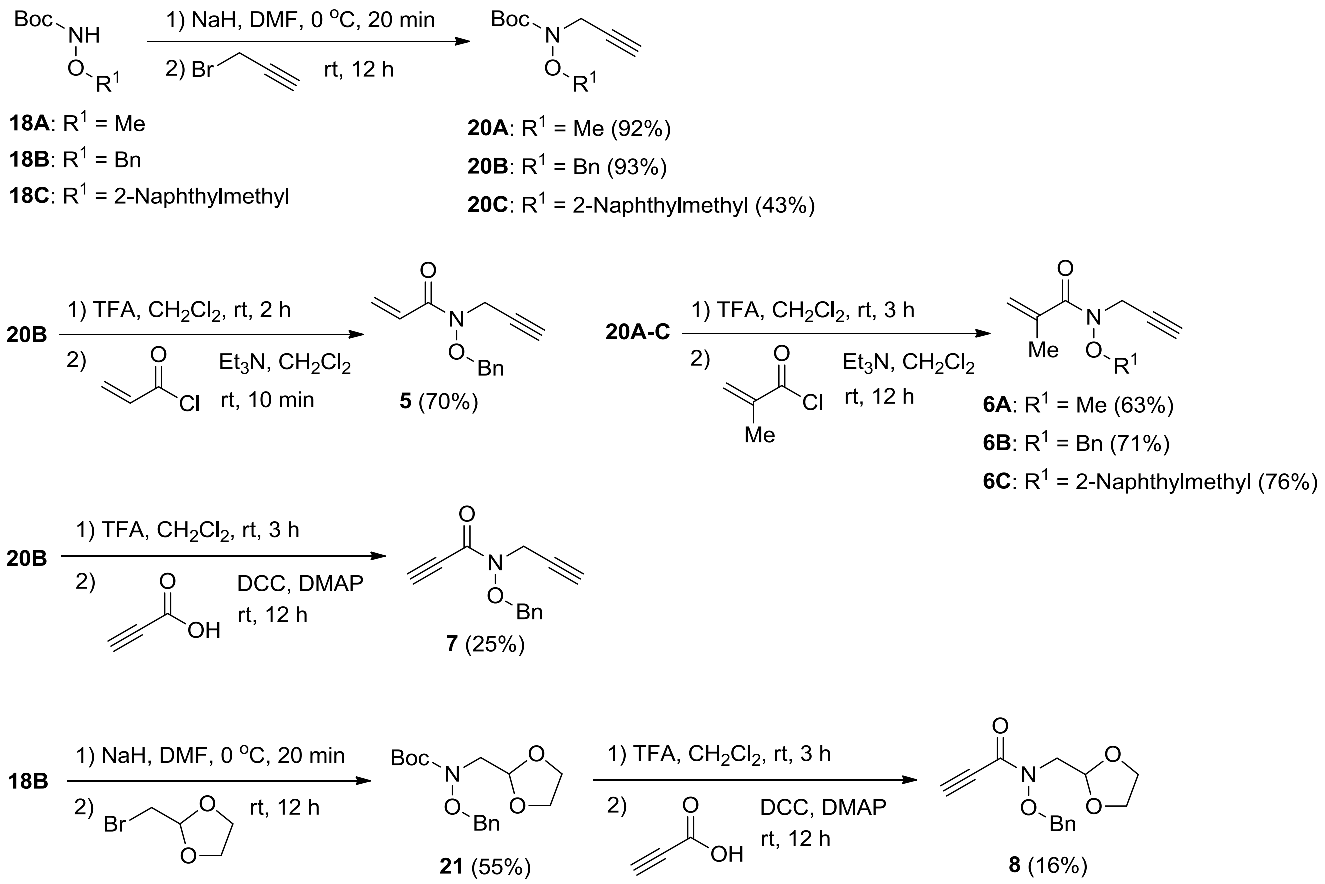


**Scheme 2**

**(20A).** Colorless oil; IR ($CHCl_3$) 1719 cm$^{-1}$; $^1$H NMR ($CDCl_3$) δ 4.18 (2H, d, *J* = 2.4 Hz), 3.77 (3H, s), 2.26 (1H, t, *J* = 2.4 Hz), 1.51 (9H, s); $^{13}$C NMR ($CDCl_3$) δ 156.2, 82.1, 78.3, 71.5, 62.9, 39.5, 28.0; MS (CI$^+$): *m/z* 186 (M + H$^+$, 0.2), 55 (100); HRMS (CI$^+$): calcd for $C_9H_{16}NO_3$ (M + H$^+$): 186.1129, found: 186.1135.

**(20C).** Colorless oil; IR ($CHCl_3$) 1706 cm$^{-1}$; $^1$H NMR ($CDCl_3$) δ 7.88-7.82 (4H, m), 7.58 (1H, dd, *J* = 8.5, 1.5 Hz), 7.52-7.47 (2H, m), 5.11 (2H, s), 4.13 (2H, d, *J* = 2.4 Hz), 2.25 (1H, t, *J* = 2.4 Hz), 1.51 (9H, s); $^{13}$C NMR ($CDCl_3$) δ 156.6, 133.3, 133.2, 133.1, 128.7, 128.2, 128.1, 127.7, 127.0, 126.3, 126.1, 82.3, 78.5, 77.8, 71.8, 40.3, 28.1; MS (FAB$^+$): *m/z* 312 (M + H$^+$, 2), 141 (100); HRMS (FAB$^+$): calcd for $C_{19}H_{22}NO_3$ (M + H$^+$): 312.1599, found: 312.1604.

**(5).** Colorless oil; IR ($CHCl_3$) 1661 cm$^{-1}$; $^1$H NMR ($CDCl_3$) δ 7.44-7.32 (5H, m), 6.67 (1H, dd, *J* = 17.1, 10.4 Hz), 6.43 (1H, dd, *J* = 17.1, 1.8 Hz), 5.74 (1H, dd, *J* = 10.4, 1.8 Hz), 5.01 (2H, s), 4.44 (2H, d, *J* = 2.4 Hz), 2.28 (1H, t, *J* = 2.4 Hz); $^{13}$C NMR ($CDCl_3$) δ 167.6, 134.2, 130.1, 129.4, 129.2, 128.8, 125.9, 78.4, 77.9, 72.2, 36.9; MS (EI$^+$): *m/z* 215 (M$^+$, 13), 92 (100); HRMS (EI$^+$): calcd for $C_{13}H_{13}NO_2$ (M$^+$): 215.0946, found: 215.0941.

**(6A).** Colorless oil; IR ($CHCl_3$) 1661, 1640 cm$^{-1}$; $^1$H NMR ($CDCl_3$) δ 5.36 (1H, s), 5.27 (1H, s), 4.35 (2H, d, *J* = 2.4 Hz), 3.75 (3H, s), 2.25 (1H, t, *J* = 2.4 Hz), 1.95 (3H, s); $^{13}$C NMR ($CDCl_3$) δ 171.8, 139.6, 118.3, 77.9, 72.0, 62.8, 37.1, 19.5; MS (EI$^+$): *m*/*z* 153 (M$^+$, 7), 83 (100); HRMS (EI$^+$): calcd for $C_8H_{11}NO_2$ (M$^+$): 153.0790, found: 153.0796.

**(6C).** Colorless oil; IR ($CHCl_3$) 1659, 1640 cm$^{-1}$; $^1$H NMR ($CDCl_3$) δ 7.90-7.80 (4H, m), 7.55-7.47 (3H, m), 5.44 (1H, s), 5.35 (1H, s), 5.15 (2H, s), 4.39 (2H, d, *J* = 2.4 Hz), 2.30 (1H, t, *J* = 2.4 Hz), 1.99 (3H, s); $^{13}$C NMR ($CDCl_3$) δ 171.9, 139.7, 133.3, 133.0, 131.9, 128.6, 128.3, 127.9, 127.6, 126.6, 126.4, 126.2, 118.3, 77.9, 77.7, 72.3, 38.3, 19.5; MS (FAB$^+$): *m*/*z* 280 (M + H$^+$, 10), 141 (100); HRMS (FAB$^+$): calcd for $C_{18}H_{18}NO_2$ (M + H$^+$): 280.1338, found: 280.1344.

**(7).** Colorless oil; IR ($CHCl_3$) 1653 cm$^{-1}$; $^1$H NMR ($CDCl_3$) δ 7.47-7.44 (2H, m), 7.42-7.32 (3H, m), 5.12 (2H, s), 4.36 (2H, br s), 3.22 (1H, s), 2.32 (1H, br s); $^{13}$C NMR ($CDCl_3$) δ 154.1, 134.0, 129.4, 129.0, 128.5, 80.0, 78.6, 76.6, 74.9, 72.7, 36.9; MS (CI$^+$): *m*/*z* 214 (M + H$^+$, 100); HRMS (CI$^+$): calcd for $C_{13}H_{12}NO_2$ (M + H$^+$): 214.0868, found: 214.0870.

**(21).** Colorless oil; IR ($CHCl_3$) 1701 cm$^{-1}$; $^1$H NMR ($CDCl_3$) δ 7.44-7.40 (2H, m), 7.38-7.29 (3H, m), 5.16 (1H, t, *J* = 4.9 Hz), 4.89 (2H, s), 3.96 (2H, m), 3.87 (2H, m), 3.59 (2H, d, *J* = 4.9 Hz), 1.50 (9H, s); $^{13}$C NMR ($CDCl_3$) δ 156.6, 135.5, 129.4, 128.3 (2C), 101.4, 81.4, 76.9, 64.8, 52.7, 28.1; MS (FAB$^+$): *m*/*z* 310 (M + H$^+$, 16), 91 (100); HRMS (FAB$^+$): calcd for $C_{16}H_{24}NO_5$ (M + H$^+$): 310.1654, found: 310.1647.

**(8).** Colorless oil; IR ($CHCl_3$) 1649 cm$^{-1}$; $^1$H NMR ($CDCl_3$) δ 7.48-7.32 (5H, m), 5.17 (1H, t, *J* = 4.6 Hz), 5.02 (2H, s), 3.99 (2H, m), 3.89 (2H, m), 3.79 (2H, d, *J* = 4.6 Hz), 3.21 (1H, s); $^{13}$C NMR ($CDCl_3$) δ 154.4, 134.1, 129.4, 128.9, 128.5, 100.4, 79.5, 77.9, 75.2, 64.9, 49.8; MS (FAB$^+$): *m*/*z* 262 (M + H$^+$, 100); HRMS (FAB$^+$): calcd for $C_{14}H_{16}NO_4$ (M + H$^+$): 262.1079, found: 262.1087.

Substrate **12** was prepared as shown in Scheme 3 and **22** is a known compound [8].

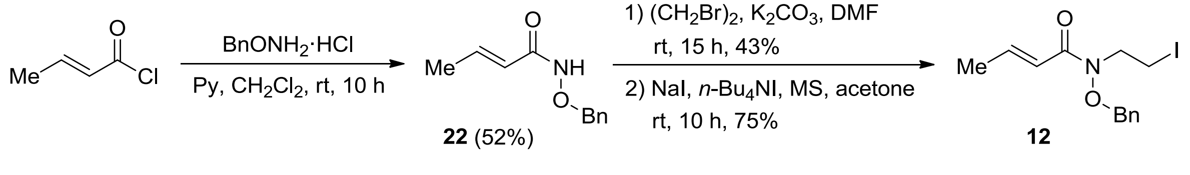


**Scheme 3**

**(22).** Colorless crystal; mp 105–109 °C (AcOEt/hexane); IR ($CHCl_3$) 1683 cm$^{-1}$; $^1$H NMR ($CD_3OD$) δ 7.45-7.27 (5H, m), 6.85 (1H, m), 5.76 (1H, br d, *J* = 15.0 Hz), 4.85 (2H, s), 1.82 (3H, d, *J* = 6.7 Hz); $^{13}$C NMR ($CD_3OD$) δ 166.2, 142.1, 137.0, 130.3, 129.7, 129.5, 122.4, 79.1, 18.0; MS (EI$^+$): *m*/*z* 191 (M$^+$, 3), 91 (100); HRMS (EI$^+$): calcd for $C_{11}H_{13}NO_2$ (M$^+$): 191.0946, found: 191.0941; anal. calcd for $C_{11}H_{13}NO_2$: C, 69.09; H, 6.85; N, 7.32; found: C, 68.81; H, 6.79; N, 7.23.

**(12).** Colorless oil; IR ($CHCl_3$) 1714 $cm^{-1}$; $^1H$ NMR ($CDCl_3$) δ 7.30-7.20 (5H, m), 6.34 (1H, m), 5.72 (1H, dd, $J$ = 15.5, 1.6 Hz), 4.92 (2H, s), 4.33 (2H, d, $J$ = 7.3 Hz), 3.19 (2H, d, $J$ = 7.3 Hz), 1.71 (3H, dd, $J$ = 6.8, 1.6 Hz); $^{13}C$ NMR ($CDCl_3$) δ 153.8, 137.3, 134.1, 128.3, 128.1, 127.9, 121.7, 76.4, 71.5, 17.9, 2.0; MS ($FAB^+$): $m/z$ 346 (M + $H^+$, 41), 91 (100); HRMS ($FAB^+$): calcd for $C_{13}H_{17}INO_2$ (M + $H^+$): 346.0304, found: 346.0302.

Substrates **14** and **16** were prepared as shown in Scheme 4. The characterization data of **14** were reported in our previously published article [3] and the characterization data of **16**, **23** and **24** in [4].

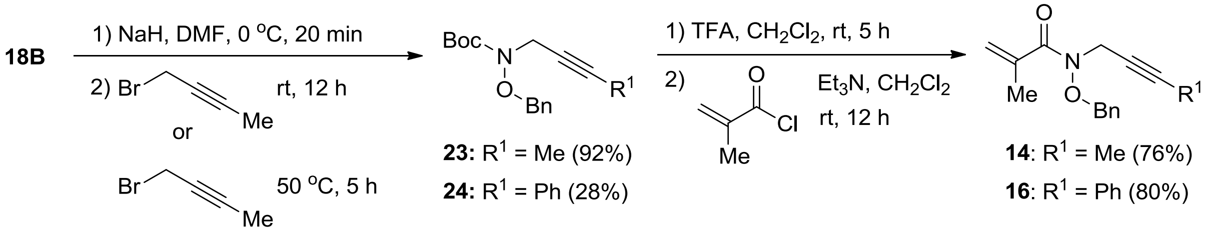


**Scheme 4**

## 4. References


1 Barnes,D. M.; Ji, J.; Fickes, M. G.; Fitzgerald, M. A.; King, S. A.; Morton, H. E.; Plagge, F. A.; Plagge, M.; Wagaw, S. H.; Wittenberger, S. J.; Zhang, J. *J. Am. Chem. Soc.* **2002**, *124*, 13097-13105.

2 Miyabe, H.; Asada, R.; Takemoto, Y. *Org. Biomol. Chem*. **2012**, *10*, 3519-3530.

3 Miyabe, H.; Asada, R.; Toyoda, A.; Takemoto, Y. *Angew. Chem. Int. Ed.* **2006**, *45*, 5863-5866.

4 Yoshioka, E.; Kohtani, S.; Sawai, K.; Kentefu; Tanaka, E.; Miyabe, H. *J. Org. Chem.* **2012**, *77*, 8588-8604.

5 Grigg, R.; Rankovic, Z.; Thoroughgood, M. *Tetrahedron* **2000**, *56*, 8025-8029.

6 Sulsky, R.; Demers, J. P. *Tetrahedron Lett.* **1989**, *30*, 31-34.

7 Caprio, V. E.; Jones, M. W.; Brimble, M. A. *Molecules* **2001**, *6*, m200.

8 Hearn, M. T. W.; Ward, A. D. *Aust. J. Chem.* **1969**, *22*, 161-173.
